# Legal Medicine

**Published:** 1824-09-01

**Authors:** 


### Legal Medicine.

1. *Wounds by Fire Arms. (Medico-Legal.)* The following curious fact is not undeserving of attention by the Medico-legal Inquirer. An old man was fired at from a deep ditch on the road-side, during a thick fog, and killed on the spot. A near relation, who was successor to his property, and whose menaces and conduct for some time previous, were of an alarming nature, was suspected of the murder, and arrested. It was proved that, a few minutes before the murder was committed, the accused was seen very close to the fatal spot, with a fowling piece in his hand. At each successive examination, the accumulation of moral evidence increased, so that no doubt was left on the minds of the jury, although there was no direct ocular witness of the murder. But the scene changed, when the *procès-verbal* of the dissection was read in court. It was proved by the surgeons, that the death had been occasioned by two balls, one of which cut the aorta across, and the other passed through the ilium. The hole in the ilium was perfectly circular, and when accurately measured, was found to be eight lines in diameter. The calibre of the prisoner's fowling-piece (the only arms in his possession) was found to be only six and a half lines in diameter. This circumstance at once set the prisoner at liberty. It was supposed that the assassin must have fired from a military musket, and escaped in the fog.

Some time after this, an old officer of the Gendarmerie committed suicide by means of a cavalry pistol. The ball perforated the parietal bone, traversed the brain, &c. The hole where it entered was perfectly circular, and, when accurately measured, was found not only to greatly exceed the calibre of the pistol, but, in fact, to admit, without much force, the barrel of the pistol itself.

The bearing of this case upon the preceding will be sufficiently manifest. We greatly fear that the moral or circumstantial evidence was right, and the physical or medico-legal evidence wrong—and if so, that science saved a murderer from the gallows. But this was better than that an innocent man should suffer. We think the jury were right in acquitting the prisoner in the case abovementioned; because, in the great majority of cases, it will be found that, when a bullet has passed through a solid substance—wood for example, the hole will be smaller than the bullet. In passing through a very hard and inelastic substance, however, we can easily conceive that the diameter of the perforation may sometimes exceed that of the perforating body, in consequence of the destruction of parts extending somewhat beyond the immediate sphere of the destructive projectile. The case we have detailed is worthy of a place in the medico-legal student's memory.—GAZETTE DE SANTE, *Janvier*, 1824.

---

2. *Poisoning by Cicuta.* Four children ate of *cicuta virosa* that grew by the side of a rivulet. Of these, three died in convulsions—the fourth was saved by an emetic promptly administered. The following were some of the principal appearances noted on dissection:—a number of bluish red spots on the skin—the pupils dilated—the vessels of the conjunctiva gorged. In the chest, the lungs, though otherwise sound, were of a bluish red colour—gorged with blood—as were the vessels of the pleura—the heart was not flaccid, and the right chambers contained much blood. In the abdomen, the stomach and intestinal canal were distended with gas. On the internal surface of the stomach the mucous membrane was found covered with brownish spots, beneath which there was an appearance of gangrene. The same were observed in the small intestines. Nothing unusual on the external surfaces of these parts. The epiglottis and pharynx were red, and much mucus in the trachea. The vessels of the brain were highly injected, as if the little patient had died of apoplexy.—*Journal Complement. Fevrier*, 1824.

---

3. *Apothecaries' Act.* It would almost appear that this act is principally calculated to annoy those for whose benefit it was designed!—The following recent trial will exemplify this remark. Mr. Malmsey, who had regularly passed Apothecaries' Hall, brought an action against Mr. Abbott for medicines and medical attendance furnished to defendant's son. A verdict was found for the plaintiff at Shrewsbury. But a counter-action was set up, and a motion made for rule to shew cause why the verdict should not be set aside, and a non-suit entered upon. It was shewn that, according to the act, no certificate is valid unless signed by a majority of the Court of Examiners, of whom eight form a quorum. The plaintiff, at the trial, produced a certificate signed by four persons, purporting to be Examiners, and counter-signed in the margin by a person calling himself Secretary to the Board. *But no proof was given that these names* (with the exception of Mr. Johnson and Mr. Wheeler) were members of the Court—and Mr. Wheeler's hand-writing alone was verified. Here then arose the objection—for the Act required that the instrument *should be proved to be signed by a majority of the Court.* The signature of the Secretary was of no avail, because the statute did not recognize such an officer. His name therefore could not authenticate a document of which the necessary legal proof was, first, that—*the hand-writing of the subscribers should be verified*—and secondly, that—*it should be shewn they formed a majority of the Court of Examiners.* Although it appeared to Mr. Justice Bayley, that the counter-signature of the Secretary *ought to be taken as good evidence of the signatures of the members,* yet the law was imperious, and the Court had no course left but to administer the law as they found it. The rule *nisi* was therefore granted.

Till some alteration be made in the Act, therefore, it is necessary for every apothecary who has passed the Hall, and who sues for five pounds reward for medicines and attendance, to bring persons into court who can prove the signatures of the Court of Examiners! To use the words of an acute medico-legal cotemporary writer, (Dr. Gordon Smith) it appears that the only apothecaries, *de jure*, are those who, *having no certificate*, can prove that they were practising as such *on* Tuesday, August the 1st, 1815!—So much for the glorious uncertainty of the law!

We will add one word of advice to our junior brethren—and that is—never to bring an action against a patient for recovery of debts. Medical remuneration should not be put on the same footing with remuneration for *work done*, or ponderable substances served out over a counter—nor should the same measures be resorted to where remuneration is withheld. The character of a litigious doctor is not less degrading or injurious than that of an avaricious one. We had rather live on bread and water than wring from the gripe of the sordid miser, or ungenerous wretch, the paltry sum which a Court might award, but which a medical man should despise. And, laying aside all views and feelings of a philosophic nature—shaping our course by the Koran of the counting-house—we are convinced that it is sound *pecuniary* policy in a medical man to avoid, *coute qui coute*, an appeal to the law for professional remuneration.

---

4. *Poisoning by Prussic Acid.* As the inspection of bodies destroyed by this subtle poison has not yet furnished unequivocal data whereon to ground a medico-legal evidence on the subject, M. Mertzdorp thinks he can contribute to the present stock of our knowledge, by the results of two dissections of suicides in which he was judicially concerned—one in the year 1819—the other in 1821.

*Case* 1. A hypochondriac, aged 44 years, swallowed, at eight o'clock in the morning, two drachms of the etherial oil of bitter almonds—threw himself back on his bed, and called to his house-keeper, who was in the next room, and who, on entering his chamber, saw his eyes roll upwards—his face spasmodically affected—and his chest heaving laboriously. In twenty minutes, a physician arrived, who found the patient insensible—his eyes open and fixed—the pupils immoveable—the breathing stertorous, and becoming slower every moment—the pulse scarcely perceptible. In ten minutes more the patient expired, his body emitting a strong odour of bitter almonds. This was on the 9th of February, 1819. *Dissection* was performed 29 hours after death, and even then the putrefactive process had far advanced, and the body was covered with numerous greenish blue spots. Nearly pure blood issued from mouth and nose whenever the body was moved; both this and the whole body, exterior and interior, exhaled, notwithstanding the putrefaction, a strong effluvium of prussic acid. The jaws were firmly locked. On opening the abdomen, the stomach and intestines were found distended with gas, and very red. The cardiac and pyloric orifices were redder than the other portions of the stomach externally. In the stomach itself were found about six ounces of a brownish fluid, smelling strongly of bitter almonds. The mucous membrane of this organ was very red, and marked with stripes of a sanguineous appearance. The small intestines presented nearly the same appearance. The hydro-cyanic odour was perceptible throughout the tube, but more strongly so as it approached the stomach. The liver was livid and very voluminous, from which a violet-coloured liquid blood flowed. The same was observable in the spleen. The blood in the kidnies was similar to that in the liver and spleen. Nothing else remarkable in the abdomen. In the thorax the lungs were found flaccid, containing little blood, but swimming in a sanguinolent fluid which filled the cavity of the pleura. The heart was also flaccid, with gas and a little blood in its cavities, Nothing else remarkable in this portion of the body. In the head the vessels were found filled with the same kind of blood as in the rest of the body, except that it had less of the odour of bitter almonds. There was a serous effusion under the arachnoid. The cerebral substance was soft.

*Case* 2. A young man, about 20 years of age, (un pharmacien) swallowed, as nearly as could be guessed, about three drachms and a half of Ittner's prussic acid. He was found dead in his bed, but the interval between swallowing the poison and dissolution could not be ascertained. The appearances on the exterior of the body were not precisely those remarked on the exterior of the other suicide, nor did the body exhale the odour of prussic acid. But the cavities presented many of the same characters as in the former case, and the hydrocyanic effluvium (not exactly that of laurel water) was perceptible in the stomach. In both, the blood was of a deep violet colour, fluid, and much accumulated in the head—in both, the stomach and small intestines were inflamed—though putrefaction had not advanced so far in the young man as in the elder.

We leave our toxicologists and jurisprudents to draw their own conclusions how far these cases are capable of guiding them in similar medico-legal examinations. It is not improbable that they may often be called upon to examine the bodies of suicides destroyed in this manner.

---

5. *Simulated Diseases.** Mr. Hutchison has related a great many curious instances of feigned diseases among our seamen, during the late protracted war, his situation, as Surgeon of the Royal Naval Hospital at Deal, having afforded him ample opportunities of witnessing, and often of detecting, such impostures. We shall glance at some of these.

1. *Ulcers.* These have been formed and kept up by first incising a piece of skin, and then applying a blister, quick lime, mineral acids, &c. In a limb which Mr. H. amputated, he found a piece of copper coin imbedded between the gastrocnemius and soleus muscles. The man confessed that he had thrust this substance into the ulcer, about nine months previously! The usual security against such tricks, is a well-applied bandage, from the toes to the knee, sealed with wax ; but this was found inefficient, as pins were thrust through it, and the ulcer irritated. A wooden box was then placed round the limb, which proved effectual in preventing such practices.

2. *Diarrhœa.* Our author has known diarrhœa, and even dysentery, induced in hospital, for the purpose of invaliding—sometimes destroying the life of the infatuated self-tormentor. The means most resorted to by our seamen, were mixtures of vinegar and burnt cork, by which some of the finest young men in the service destroyed themselves.

3. *Fever.* This is a disease which Dr. H. has known to be feigned. He relates a case where a French prisoner swallowed tobacco, and covered his tongue with soap. The tobacco caused great rapidity of the pulse, but the matters ejected from the stomach smelled so strongly of tobacco, that the imposition was soon detected, and confession followed.

4. *Contractions of Hands, Elbows, Knees, &c.* These are very frequently feigned by sailors. They can only be detected by carefully attending to a variety of circumstances, and taking the impostors off their guard.

5. *Ophthalmia,* has been produced by putting irritating substances into the eyes, as alum, lime, tobacco, &c.

6. *Incontinence of Urine.* The best mode of detecting this imposture, in our author's experience, was the exhibition of laudanum, unknown to the person, and, when fast asleep, to place a clean napkin

---

* Mr. Hutchison, Med. and Phys. Journal, February 1824.

under him. The napkin, of course, will remain dry during sleep, if the disease be feigned, but be soiled, if real.

7. *Strictures.* This disease was often feigned by naval officers, when they wanted to leave their ships, from any cause of dislike to the captain or others. Mr. Hutchison generally detected it by placing the person against a wall, so as not to admit of retreat. A bougie was then passed into the urethra, when, in many cases, much difficulty was experienced in getting it beyond the perineum. If suspicion of imposture be entertained, the mind of the patient is to be drawn off, if possible, and an opportunity taken of passing the bougie into the bladder.

Are we not liable to some error here, when spasmodic stricture exists, which may allow the bougie to pass occasionally, though it may be a very troublesome complaint to the patient in general.

8. *Hernia.* Mr. Hutchison remarks that, although seamen are very liable to ruptures, from their great exertions, in furling sails, heaving up anchors, and working the great guns, yet the operation for strangulated hernia, is very seldom necessary. This we know to be the case; but then, we think the same remark extends to private life. There is not, we think, one case in 5000, which requires the operation, throughout England. Seamen, too, have *immediate* medical assistance, when an accident happens, which, of course, diminishes the chance of serious results.

Mr. H. concludes this paper with a curious instance of voluntary power over the cremaster muscles, possessed by a man who could draw the testes up into the groin, so as to produce the appearance of hernia there, and afterwards let them drop down to the bottom of the scrotum, when the examination was over. Mr. Hutchison, was, however, too lynx-eyed an observer to be deceived by this; for, although the tumours in the groin puzzled him at first sight, he soon proved an *alibi* of the testicles from their proper domicile in the scrotum, and caught them peeping through the Pope's eyes. The man, on being detected, acted like a philosopher, "and seeing no longer any chance of eluding the King's service, displayed before us several very remarkable feats of power he possessed over these organs."

Mr. H. promises a continuation of his paper, which is both amusing and instructive.

---

6. *Medical Remuneration.* The general practitioners are greatly to blame not to unite for the purpope of devising some plan of changing the present disrespectable mode in which they are remunerated for their services. We are quite convinced that they are every day losing ground in public estimation; or, at least, that the public antipathy to their *practice* is daily on the increase. If they resolutely persist in their present course, they will, in a few years, throw almost the whole of their practice (in large towns especially) into the hands of physicians, prescribing surgeons, and dispensing chemists. This is as sure to ensue as the sun is to rise to-morrow morning. Physicians and surgeons are now becoming so numerous, that a considerable portion of the younger branches will attend at a much lower rate than the regular fee claimed by the seniors; thus accommodating the various classes of society. And even if this were not to be the case, the public are becoming so squeamish about swallowing large quantities of medicine that they will give the guinea for the prescription, rather than bring in the general practitioner and commence another long bill. This proves, that people will give much money for little medicine, rather than little money for much medicine. It ought to prove a lesson to the apothecary. Let him state fairly to his patients, that he will send as little medicine as possible, and charge half-a-crown or three shillings for his visit, appealing to their good sense and feeling which plan to accept. We know, from the practice of many large provincial towns, that this might be done. In several of these last, the items are never made out, unless the patient insists upon it, which is hardly ever the case. It is to be taken into consideration, that there are three things on the increase at present—wealth, luxury, and knowledge. All and every of these are in favour of large fees, and small doses of physic. But this by the way.

In the Court of King's Bench, a trial lately came on, for recovery of an apothecary's bill, in the case of Cole *versus* Devereux. The charges were proved to be reasonable by respectable witnesses; but still, the defendant's counsel shewed that boxes of pills, some of which were charged *3s. 6d.* had been sent, sometimes, twice in one day. This is an example of the misery of making the whole charge to fall on the medicine alone. Thus, if a whimsical patient, and there are many such, takes an antipathy to any form but pills, the general practitioner is forced to send either an enormous number of these, or charge an exorbitant price for each box. Mr. Cole recovered about two thirds of his bill.

---

7. *Case of Recovery from supposed punctured Wound of the Diaphragm.** Thomas Rowbotham, ætat. thirty-five, received two wounds in the abdomen, from a shoemaker's knife, on Friday, 28th of June, 1816. The blade of the instrument, about three inches long, and sharp-pointed, was plunged up to the handle into the body. The wounds, one on the right, the other on the left side of the epigastrium, bled profusely—the more so, as he was obliged to walk nearly a quarter of a mile directly afterwards. On the day following, the right wound still bled freely, and at intervals, on Sunday and Monday, with the emission of air bubbles, resembling those produced by vinous fermentation. As the hæmorrhage subsided, considerable extravasation of blood, with emphysema, appeared gradually extending over the abdomen, which, on Tuesday, was much swollen, with a degree of tension. From the left wound, there was some bleeding; at times he laboured under a spasmodic difficulty of breathing, the skin yellow, and slight pyrexia,

* Mr. Wood, Med. Repos. No. 7, New Series.

at evening—depletion was fully employed, with good effect. On the following Sunday, suppuration was established, and he was able to sit up—in a fortnight, the wounds were healed, and no complaint but debility remained.

*Commentary.* We do not think that the chest was penetrated by either of the above wounds. An instrument three inches in length, plunged into the *left* epigastrium, would have to traverse the left lobe of the liver (position perpendicular) before it entered the chest—and if it did enter the chest, it would, most probably, be where the pericardium is attached to the diaphragm. In this case, we conceive, the symptoms would have been different.

In respect to the wound in the *right* side of the epigastrium, an instrument three inches in length, plunged in, and slanting upwards (position as before) would inevitably come in contact with the liver—and we think such an instrument could not penetrate the chest in that side. It appears from the icteritious symptoms, that the liver was actually wounded, in this case. As for the bubbles of air, and emphysema of the abdomen, such phenomena would be more likely to take place, under the above circumstances, from puncture of the colon, or other intestine, than wound of the lungs.

---

8. *Defamation of Character.* A medical practitioner, of the name of Pierpoint, residing in Worcester, lately brought an action against a lady there, for aspersing his character. He had administered an emetic to a Mrs. Isaac, who died some time afterwards, and the practice was loudly condemned by the sister of the patient, who used very improper language on the occasion. A verdict was given in favour of Mr. Pierpoint, and thirty-nine shillings damages. We mention this trial, because we are not quite satisfied with the evidence of one of the medical witnesses, Dr. Lewis, who appears never to have seen the patient, dead or alive, condemned the exhibition of the emetic, as injudicious. The evidence of Dr. Johnson, of Birmingham, was a model of medical ethics, upon this occasion, and, therefore, we shall quote it. "The expediency," says this veteran physician, "of administering an emetic, must depend on the *symptoms of the moment.* If called in, when Dr. Malden was, I could not tell what the symptoms had been on the 10th of April, *nor judge of the expediency of administering an emetic.* If the patient felt relieved (which it was proved she did) I could not ascribe the symptoms of inflammation afterwards appearing to the emetic." This was the just, the honorable, the ethical, and, we may add, the truly Christian evidence to give. We forbear to set a name on any other kind of evidence in similar cases.

We know nothing of Mr. or Dr. Pierpoint. The *man* has nothing to do with the *ethics* of the question. All personal considerations should be excluded from the mind of a witness, on such occasions—and, certainly, we shall not fail to stigmatize any deviation from the rule of right, on the part of our professional brethren, when called in to pass judgment on the conduct of their brother practitioners.

